# Mona Lisa is always happy – and only sometimes sad

**DOI:** 10.1038/srep43511

**Published:** 2017-03-10

**Authors:** Emanuela Liaci, Andreas Fischer, Markus Heinrichs, Ludger Tebartz van Elst, Jürgen Kornmeier

**Affiliations:** 1 Institute for Frontier Areas of Psychology and Mental Health, Freiburg, Germany; 2 Eye Center, Medical Center, University of Freiburg, Freiberg, Germany; 3 Center for Mental Disorders, Medical Center, University of Freiburg, Freiberg, Germany; 4 Faculty of Medicine, University of Freiburg, Germany; 5 Laboratory for Biological and Personality Psychology, Department of Psychology, University of Freiburg, Freiberg, Germany

## Abstract

The worldwide fascination of da Vinci’s Mona Lisa has been dedicated to the emotional ambiguity of her face expression. In the present study we manipulated Mona Lisa’s mouth curvature as one potential source of ambiguity and studied how a range of happier and sadder face variants influences perception. In two experimental conditions we presented different stimulus ranges with different step sizes between stimuli along the happy-sad axis of emotional face expressions. Stimuli were presented in random order and participants indicated the perceived emotional face expression (first task) and the confidence of their response (second task). The probability of responding ‘happy’ to the original Mona Lisa was close to 100%. Furthermore, in both conditions the perceived happiness of Mona Lisa variants described sigmoidal functions of the mouth curvature. Participants’ confidence was weakest around the sigmoidal inflection points. Remarkably, the sigmoidal functions, as well as confidence values and reaction times, differed significantly between experimental conditions. Finally, participants responded generally faster to happy than to sad faces. Overall, the original Mona Lisa seems to be less ambiguous than expected. However, perception of and reaction to the emotional face content is relative and strongly depends on the used stimulus range.

Although our daily perceptual experience appears to us as an unambiguous and stable reflection of the world, the sensory information is inherently incomplete and ambiguous due to the limited capacity of our senses^1^. In order to overcome this natural limitation, the human brain has to disambiguate the sensory information and construct meaningful interpretations.

Ambiguous figures, like the famous Necker Cube^2^, are extreme cases, where the sensory information is maximally ambiguous, making our perception unstable, alternating spontaneously between the two most probable interpretations. This happens repeatedly, even though the physical properties of the stimulus, and its retinal projections, remain unchanged. Examples for such perceptual instabilities, resulting from ambiguous sensory input, exist at different levels of complexity, like low-level geometry (Necker Cube), figure-ground ambiguity (Rubin’s Vase-Face stimulus^3^), motion (e.g. von Schiller’s Stroboscopic Alternative Motion stimulus, “SAM”^4^,^5^), and also at more complex semantic levels (e.g., Boring’s Old/Young woman^6^,^7^). Ambiguity even exists in other modalities like audition and touch^8^. Numerous studies focused on behavioral and physiological correlates of perceptual processing of such ambiguous figures^9^,^10^,^11^.

Sensory ambiguity and perceptual instability can also be found at even more complex levels, e.g. in language^12^ and, closely related, during non-verbal aspects of communication, when the emotional content of face expressions has to be interpreted^13^. Indeed, configurations of facial muscles appear to be inherently ambiguous^13^, and in order to resolve this ambiguity and understand a facial emotional expression, the information from the face must be combined with information from speech^14^ and other sensory modalities^15^,^16^. One famous example of ambiguity in emotional face expressions – as discussed by experts – is Leonardo da Vinci’s (around 1503–1507) Mona Lisa painting (Fig. 1). As the English essayist and writer Walter Pater affirmed in a prominent essay, dedicated to Leonardo da Vinci^17^, Mona Lisa’s smile holds an “emotional ambiguity”, revealing first a “promise of an unbounded tenderness”, but soon after also a “sinister menace”. Her expression is worldwide accepted to be an emblem of emotional ambiguity. The notable English art historian Ernest Gombrich wrote in “*Story of Art*”^18^, that “sometimes she seems to mock at us, and then again we seem to catch something like sadness in her smile”.

During the recent years, the elusive quality of Mona Lisa’s painting has been object of scientific investigations^19^,^20^. So far all studies – to our best knowledge – take Mona Lisa’s emotional face expression as a priori ambiguous. The aim of the present study was to quantify the effective degree of ambiguity of da Vinci’s painting along the happy-sad axis of emotional expressions by applying a variant of the method of constant stimuli^21^, a well-established psychophysical method to estimate perceptual thresholds.

The facial feature, which determines the most mysterious and ambiguous character of Mona Lisa’s expression, has been found to be the mouth area^19^,^20^,^22^. We created a number of Mona Lisa’s variants by manipulating the curvature of the mouth in a systematic manner, in order to stepwise disambiguate them towards happy and sad face expressions.

To our great surprise the stimulus corresponding to da Vinci’s original painting was almost always perceived as unambiguously happy.

In a second experimental condition (Half-Range Condition) we reduced the range of face expressions, taking da Vinci’s variant as the most unambiguously happy face and additionally decreasing the step size between sadder face variants, in order to increase the “resolution of emotional ambiguity”.

The happiness bias of da Vinci’s original remained with this smaller range of emotional faces, but the resulting psychometric function differed from the first condition (Full-Range Condition). This indicates that the range of emotional face expressions strongly influences the perception of the individual face. The experiments described above had been aimed as pilots for a subsequent EEG study. Based on the surprising results we replicated these experiments in a more systematic manner. The here presented results confirm our earlier findings.

## Methods

### Participants

Twelve observers (5 males, 7 females; age range = 20–33, mean age = 26 years) participated to the experiment. Nine participants were right-handed and three left-handed. All participants were naive as to the specific experimental question and gave their written informed consent. Eleven participants reported any history of neurological disease. One participant suffers from periodic migraine but was free of symptoms during the experiments. Visual acuity was tested with the Freiburg Visual Acuity Test^23^. Eleven participants had a normal or corrected-to-normal vision. Visual acuity of one participant was 0.5 at the right eye and 0.55 at the left eye. We repeated the below analysis without this participant. Since the effects remained, we decided to keep this participant in the analysis. The study was approved by the ethics committee of the University of Freiburg and performed in accordance with the ethical standards laid down in the Declaration of Helsinki^24^.

### Stimuli

We used a grey-scale version of Leonardo da Vinci’s Mona Lisa^25^ and created 12 variants thereof by a stepwise manipulation of the curvature of Mona Lisa’s mouth in order to manipulate the emotional face expression from happy to sad. Furthermore we had to do tiny adjustments of the cheek’s shadow between stimulus variants in order to harmonize the mouth’s manipulation to the total facial expression.

**Figure 2:**
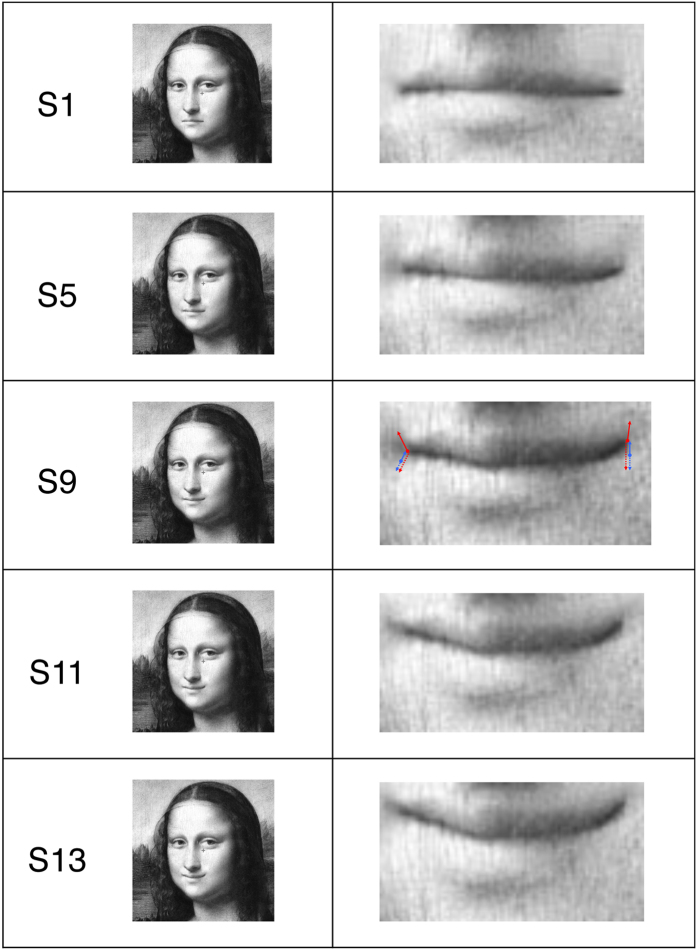
Depicted are five Mona Lisa’s variants^25^ (left column, S1, S5, S9, S11 and S13, created in Dr. Kornmeier’s lab) and the enlarged corresponding mouth regions (right column). The red and blue filled circles in S9 indicate the left and right mouth corners of the central stimulus in the Full-Range Condition (S9) and in the Half-Range Condition (S5). Arrows indicate the corresponding trajectories of the mouth corner locations of the different stimulus variants for the happy (upwards, solid lines) and sad (downwards, dashed lines) face expression.

The two experimental conditions differed in the ranges of presented stimulus variants (see Procedure for details). Figure 2 displays several Mona Lisa variants with the corresponding focus on the manipulated mouth region. Red (Full-Range Condition) and blue (Half-Range Condition) arrows on the mouth region of Da Vinci’s original version (S9) indicate the trajectories on which the left and right mouth corners of the different stimulus variants were located in the two experimental conditions. The starting points of the arrows (filled circles) mark the left and right corners of the mouth of stimulus S9 (red circles for the Full-Range Condition) and stimulus S5 (blue circles for the Half-Range Condition). Stimulus S9 (da Vinci’s original) and stimulus S5 are positioned in the middle of the two ranges from the saddest to the happiest face expressions in the Full-Range Condition and the Half-Range Condition respectively. The arrow end points mark the most extreme mouth corner positions of the Mona Lisa (“ML”) variants with the most sad and most happy face expressions for the two conditions. Dotted arrows indicate manipulations towards sad face expressions, whereas continuous arrows indicate manipulations towards happy face expressions. In both conditions we used four equally sized steps along each of the four trajectories from one stimulus variant to the next.

### Procedure

The experiment consisted of two conditions. In the Full-Range Condition we presented nine stimulus variants with roughly equidistant steps of mouth-manipulation from the saddest to the happiest stimulus variants. In the Half-Range Condition we again presented nine stimuli with equidistant steps, but decreased the range of stimulus variants and step sizes between individual stimuli to 50% of the Full-Range Condition respectively, in order to increase the “ambiguity resolution”. Five stimuli from the Half-Range Condition were also used in the Full-Range Condition.

Each experimental condition consisted of 30 blocks. In each block we presented a sequence of nine ML variants, ranging from the happiest to the saddest emotion. The stimulus order within each condition was randomized across blocks and participants. The two conditions were presented to the same group of subjects (within-subject design). All 30 repetitions per condition were executed in succession. The order of the two conditions was counterbalanced across participants.

**Figure 3:**
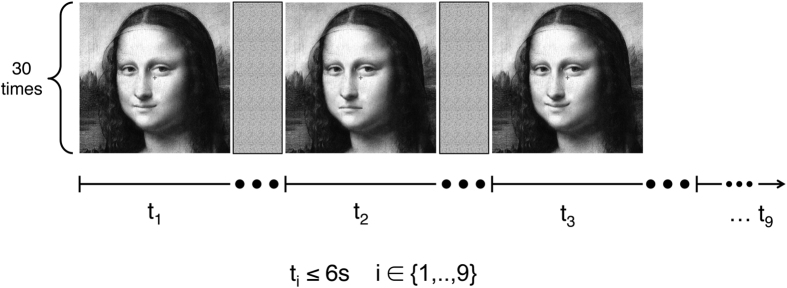
Experimental paradigm. Each of the 9 Mona Lisa variants^25^ in a block was presented for maximal 6 seconds. Within this time window the participants were told to first indicate the perceived emotional facial expression of Mona Lisa (“Perception Task”) and to subsequently rate the confidence of their response (“Confidence Rating Task”). After the second response (or after 6 s in case of no response) the next stimulus variant appeared. Each block was repeated for 30 times and the presentation order within block was randomized. Happy and sad variants of the original Mona Lisa painting were created in Dr. Kornmeier’s lab.

The participants were seated in a chair in a dimly lit room at a distance of 114 cm from the screen and observed a series of face stimuli. The average of the luminance across five image points was 64.07 cd/m^3^. In a dual task paradigm stimuli were presented for a self-paced duration, which was immediately interrupted after the second of two necessary responses, but which lasted maximally 6 s in the case of missing responses. Participants first indicated in a forced-choice manner either happy or sad face perceptions or non-face perceptions by pressing one of 3 keys (“*Perception Task*”). Subsequently they estimated the confidence of their previous response on a scale between 1 (very unsure) and 4 (highly sure) by pressing one of four different keys (“*Confidence Rating Task*”). The participants’ second response started a blank screen gap of 400 ms, followed by the next stimulus (as seen in Fig. 3).

Before the start of the main experiment, the participants performed a training part, where they learned the association between keys and face expressions. In the training, we only presented the two most disambiguated versions of ML (i.e. the saddest – S1 – and the happiest – S13 – variants). This training finished, after participants had reached a threshold of at least 8 correct responses in a series of ten stimulus presentations. The training sessions lasted for about 7 minutes.

### Analysis

#### Perception Task

**Figure 4:**
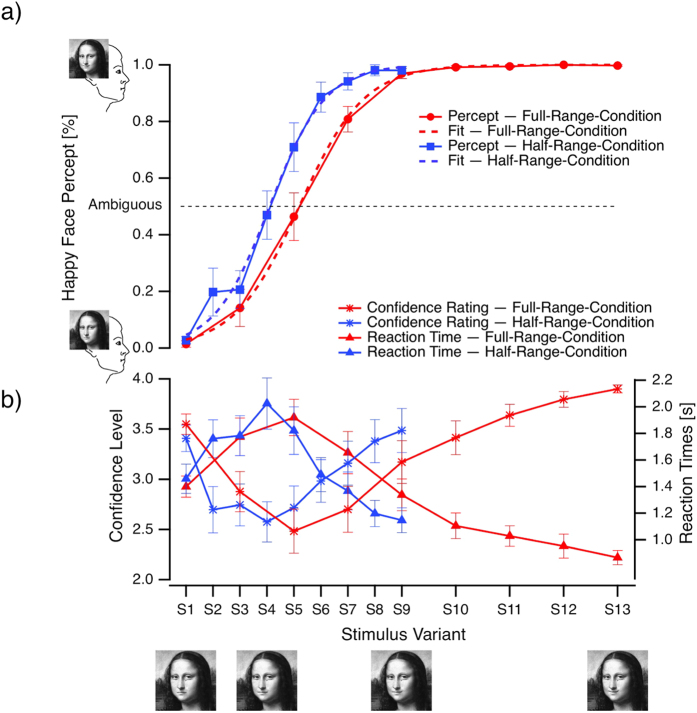
Grand mean probability of happy face percepts (Task 1, dashed traces) ± SEM (ordinate) and sigmoidal fits (continuous traces) from the Full-Range Condition (red traces) and the Half-Range Condition (blue traces) as functions of the mouth curvatur of the different Mona Lisa stimuli (abscissa). (**b**) The related reaction times (Task 1, triangles, ordinate on the right) and participants confidence ratings (Task 2, stars, ordinate on the left) of their perception responses. Happy and sad variants of the original Mona Lisa image^25^ (S9) were created in Dr. Kornmeier’s lab. Differences between sigmoid functions (**a**) are clearly visible and correspond to the differences in reaction time and confidence level traces (**b**).

For each participant and stimulus variant we calculated the percentage of happy face percepts (number of happy face responses divided by the total number of responses in the perception task). The face stimuli, which had been presented in a random order, were then numbered in increasing order from the saddest to the happiest variant and participants’ responses were sorted with respect to this order. We then fitted psychometric functions (formula 1) to the resulting response traces (see Fig. 4) and determined the stimulus number of the most ambiguous stimulus, “*S*_*amb*_” at the 50% response level (half-maximum, i.e. the sigmoidal inflection point with equal probability of happy and sad face percepts) and the *slope* of the sigmoid. The *base* and *max* values of each individual sigmoid fit were set to 0 and 1 respectively.


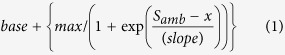


Goodness of fit was determined individually by calculating R^2^ values, which were above 90% for all participants. We thus used individual *S*_*amb*_ and *slope* values for statistical comparison of participants’ responses between the two conditions with t-tests.

Our results concerning perception of Mona Lisa motivated the execution of two additional ANOVAs. Here we further compared within each experimental condition the average *S*_*amb*_ and *slope* values from the first five trials with the average values from the last five trials using repeated-measures ANOVAS with the factors CONDITION (two steps, Half-Range and Full-Range) and PERIOD (2 steps, average of the first five and average of the last five trials) and the variables *S*_*amb*_ and *slope.* These exploratory additional analyses were not included into the repeated measures correction.

#### Confidence Rating Task

We calculated the mean confidence rating per participant and stimulus variant and entered these values into a repeated-measures ANOVA with polynomial contrasts with the factors CONDITION (two levels) and STIMULUS (five levels, focusing on the stimuli S1, S3, S5, S7 and S9 that were common to both conditions). Wilcoxon signed-rank tests were conducted for post-hoc tests.

#### Reaction Times

Reaction times were calculated as the time from stimulus onset until participant’s first response (Task 1 = Perception Task). We calculated separately for the two conditions and for each stimulus variant the mean reaction times per participant and entered the values in a repeated-measures ANOVA with polynomial contrasts with the factors CONDITION (2 levels) and STIMULUS (five levels, corresponding to the stimuli S1, S3, S5, S7 and S9, which were common to both conditions). Wilcoxon signed-rank tests were conducted for post-hoc tests.

#### Correlation between Confidence Ratings and Reaction Times

For each condition we calculated Pearson and Spearman correlation coefficients across stimulus variants between grand mean reaction times and grand mean confidence ratings.

Correction for multiple testing was applied with Holm’s variant of the Bonferroni correction^26^. In Holm’s procedure, all calculated p-values are sorted from the lowest to the highest. The first p-value is compared with an alpha corrected by the total number n of pairwise comparisons. The second p-value is compared with an alpha corrected by n-1, and so on for the following p-values. P-values that survived multiple testing corrections are reported.

## Results

### Perception of Emotional Face Expression

Within each condition, the perception of Mona Lisa’s emotional expression did change across variants describing a sigmoidal function of the percentage of happy face percepts, as shown in Fig. 4. For the Full-Range Condition, the average goodness of fit across participants was 0.999 ± 0.001 (thus about 100% of the variance was explained by the fit function). The average goodness of fit for the Half-Range Condition was 0.979 ± 0.018.

The sigmoid fit functions differed significantly between Conditions, with the location of the most ambiguous stimulus *S*_*amb*_ in the Full-Range Condition being close to S5 whereas in the Half-Range Condition *S*_*amb*_ was located close to S4 (p = 0.006, t-test, see also Fig. 4a). Further the sigmoidal fit function in the Half-Range Condition showed a steeper *slope (mean slope* = 0.67) than the fit function in the Full-Range Condition (*mean slope* = 0.87; p = 0.025, t-test, see also Fig. 4).

**Figure 5:**
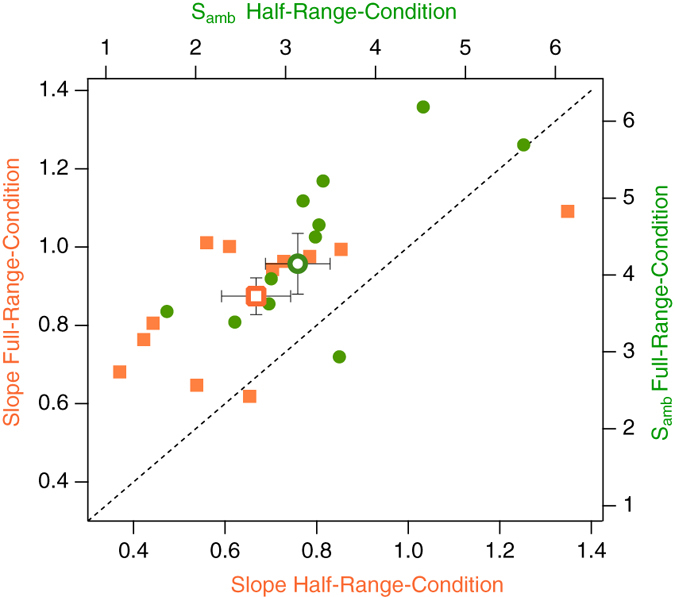
Scatter plot of the sigmoidal fit functions’ parameters for the individual participants. Orange squares: *slope* values. Green circles: *S*_*amb*_ values. Open icons indicate grand means ± SEMs. For the majority of participants, *slopes* and *S*_*amb*_ icons are above the black dashed bisection line indicating larger values for the Full-Range Condition than for the Half-Range Condition.

The present results are supported by Fig. 5, which depicts *S*_*amb*_ and *slope* values of the individual participants for the Full-Range Condition and the Half-Range Condition. Ten out of twelve participants show larger *S*_*amb*_ and *slope* values in the Full-Range Condition compared to the Half-Range Condition (the respective icons in Fig. 5 are above the bisection line).

**Figure 6:**
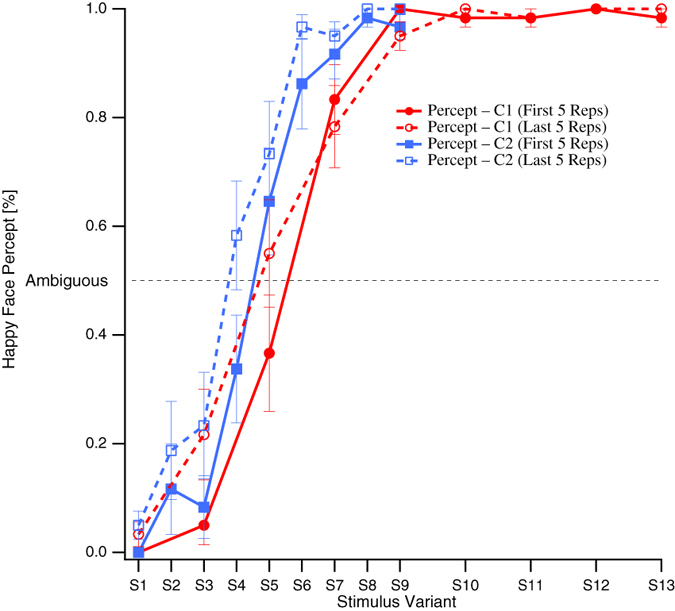
Grand mean probability of happy face percepts (Task1, ordinate) from the first 5 (solid traces) and last 5 (dashed traces) repetitions ± SEM from the Full-Range Condition (red traces) and the Half-Range Condition (blue traces) as functions of the mouth curvature of Mona Lisa variants (abscissa). Sad and happy variants (S1–S8 and S10–S13) of the original Mona Lisa image (S9) were created in Dr. Kornmeier’s lab. C1 = Full-Range Condition; C2 = Half-Range Condition; Reps = Repetitions.

ANOVA comparisons of the first with the last five trials within conditions revealed a highly significant effect for the factor CONDITION (F_(1,11)_ = 9.58, p = 0.005, uncorrected) concerning the variable *S*_*amb*_. We found a week tendency for an effect for the factor PERIOD concerning the variable *Slope* (F_(1,11)_ = 1.4, p = 0.06) but no other significant effect (see also Fig. 6).

### Confidence Rating

Perceptual confidence rating traces indicate an U-shape function with a decrease from the most unambiguous sad face stimulus towards the most ambiguous variant and an increase from the most ambiguous face stimulus towards the most happy face variant. This is indicated in the repeated-measures ANOVA as a significant quadratic effect for the factor STIMULUS (F_(1,11)_ = 43.04, p < 0.001).

The left halves (half-ranges of sad face variants) of the two confidence rating traces mainly overlap, whereas the right half-trace (half-ranges of happy face variants) of the Full-Range Condition is shifted to the right, compared to the Half-Range Condition. This is reflected in different locations of the trace minima (lowest confidence ratings) between the Full-Range Condition (around S5) and the Half-Range Condition (around S4) and indicated in the ANOVA by a significant linear interaction between STIMULUS and CONDITION (F_(1,11)_ = 8.28, p = 0.015).

Interestingly, the emotional content of the saddest S1 face and the happiest S13 face in the Full-Range Condition were both identified with close to 100% probability, however their confidence ratings differed, with higher values for the happiest than saddest emotional face variants. An exploratory post-hoc Wilcoxon signed-rank test indicated that this effect is significant (p = 0.0068).

### Reaction Times

Reaction times from the perception task (Task 1) showed inverted U-shapes for both conditions, with increasing values from the most unambiguous sad face stimulus towards the most ambiguous variant and decreasing values from the most ambiguous face stimulus towards the happiest face variant. This is indicated in the repeated-measures ANOVA as a significant quadratic effect for the factor STIMULUS (F_(1,11)_ = 32.65, p < 0.001).

Similar to the confidence ratings, the left halves of the reaction time traces (RTs to the sad face expression) overlap whereas the right half trace of the Full-Range Condition is shifted to the right, with a maximal reaction time of 1340 ms at stimulus S5, compared to the Half-Range Condition with a maximal reaction time value of 2025 ms at stimulus S4. This is supported by a significant linear interaction between STIMULUS and CONDITION (F_(1,11)_ = 7.22, p = 0.021).

Also in parallel to the findings from the confidence rating, we noticed faster reaction times for the happiest face (S13) compared to the saddest face (S1) in the Full-Range Condition. A related exploratory post-hoc Wilcoxon signed-rank test indicated that this effect is significant (p = 0.0013).

### Correlations between Confidence Ratings and Reaction Times

We found a significant negative correlation (Pearson and Spearman) between the grand mean reaction times and confidence ratings both in the Full-Range Condition (*r*_*Pearson*_ = −0.94 with p = 0.0013, *r*_*Spearman*_ = −0.93) and the Half-Range Condition (*r*_*Pearson*_ = −0.941 with p = 0.0014, r_Spearman_ = −0.9). Participants took more time for less reliable emotional face percepts (Fig. 4b).

## Discussion

One of the most often described and discussed feature of Leonardo da Vinci’s Mona Lisa painting is her ambiguous emotional face expression. In the present study we quantified for the first time Mona Lisa’s ambiguity along a happy – sad axis of emotional face expressions. We presented a copy of the original Mona Lisa and variants with stepwise increasing sadness and happiness with the following results: (1) The original Mona Lisa was always perceived as happy, whereas the most ambiguous stimulus variants had a more prominent downturn of the mouth curvature, compared to Da Vinci’s original. (2) Decreasing ambiguity of the emotional face expression in either happy or sad direction increased identification rates (from chance level towards almost 100%), reaction times (by up to factor 2) and confidence rates (by up to factor 1.5). (3) The happiest stimulus variant was identified faster and with higher confidence rates than the saddest variant, despite equal identification rates for both variants close to 100%. (4) Decreasing the range of stimulus variants caused a shift of the psychometric functions of perceived happiness. As a consequence, the perception statistics of some intermediate stimulus variants differed between conditions. This indicates that the overall stimulus range within the experimental conditions determined perception of the individual.

### Stimulus manipulation

Livingstone discussed the role of image statistics for the perception of Mona Lisa’s emotional face expression. Her image manipulations showed that low-pass filtered images uncover cheerful faces, whereas the high frequencies promote sad face expressions^22^. Kontsevich and Tyler^20^ found that the mouth region has a central role for the perception of Mona Lisa’s face expression. In the present study we restricted our stimulus manipulation to a parametric change in mouth curvature in order to create different Mona Lisa variants along the happy-sad axis of emotional face expression.

### Happy faces can be identified faster and with more confidence

In the Full-Range Condition with the larger range of stimulus variants we found faster reaction times and higher confidence ratings for the happiest compared to the saddest Mona Lisa variants. Such a reaction time difference was also visible as a tendency in the Half-Range Condition. This finding confirms previous studies, showing that observers recognize positive facial expressions faster than negative expressions^27^,^28^,^29^. An innate happy-face advantage for facial emotional recognition is discussed as possible explanation^28^ for this effect.

### Mona Lisa is always happy…

Several experts from art and history of art have discussed the fascination that emanates from da Vinci’s painting as a result of the inherent emotional ambiguity^17^,^18^. The present study tested this ambiguity for the first time by quantifying it with the well-established method of constant stimuli^21^. To our great surprise all of our participants identified the original Mona Lisa variant as happy. However, for interpretations of the current findings, one has to keep in mind the following limitation. We restricted our focus to one emotional dimension, namely the happy – sad axis of emotional face expressions. However, the “space” of emotions and emotional face expressions has more dimensions^30^ that may contribute to Mona Lisa’s ambiguity. Further our three-alternative forced-choice paradigm (happy, sad and non-face percepts) filters out any intermediate perception or any other perceptual aspects (e.g. neutral face percepts) than the binary happy vs. sad decision. Despite these limitations, our results clearly indicate that positive emotions prevail the perception of Mona Lisa.

### … but only sometimes sad

The present two experimental conditions differed in the extent of emotional face expressions with a twice as large range in the Full-Range Condition than in the Half-Range Condition. With the Half-Range Condition we aimed to identify more precisely the most ambiguous Mona Lisa variant by reducing the range and concurrently decreasing the step size between variants, thus increasing the “ambiguity resolution”. However, the smaller range did not increase the resolution of ambiguity, but instead changed the psychometric function of perceptual identification. Consequences of this were, for example, that stimulus variant S5 was identified as most ambiguous in the Full-Range Condition, but the identical stimulus was identified as happy in about 70% of the cases in the Half-Range Condition (see Fig. 4a). Further, the most ambiguous stimulus variant in the Half-Range Condition was S4. However, S4 would have been less often rated as sad in the Full-Range Condition (about 25%), as predicted by the corresponding psychometric function.

### About the potential role of adaptation

A huge amount of literature provides evidence for visual aftereffects, like priming and adaptation (for a specific example in the context of classical ambiguous figures see refs 31 and 32). In the case of adaptation, the focused observation for several seconds of an adapting stimulus containing certain stimulus features, biases perception of the subsequent test stimulus towards the opposite of the adaptation stimulus. Earlier adaptation studies showed effects for lower-level stimulus features like colour, contrast, orientation, size or motion^33^. More recent studies demonstrated very similar adaptation effects for high-level stimuli like faces, their identity, gender, ethnicity or emotional expression^33^,^34^,^35^.

Can adaptation explain the difference between the psychometric functions found in the present experiment? The present paradigm deviates fundamentally from typical adaptation paradigms: (1) Our maximal possible stimulus presentation duration is 6 s. However, the average observation time ranged between one and two seconds (see reaction times in Fig. 4). Although this is enough to reach some degree of adaptation^36^, it is shorter than typical times for full face adaptation effects (between 15 and 20 s^35^). This takes the adaptive power of our experimental procedure into question. (2) Typical adaptation experiments used one certain adaptation stimulus and one or more test stimuli within an experimental block. Each combination of adaptation and test stimuli was then repeated several times in order to get enough data for statistics. In the present experiment a series of nine Mona Lisa variants was presented in an order that was randomized between experimental blocks and participants. Therefore, any adaptation effect of a perceived stimulus by its precursor must be averaged out.

However, adaptation mechanisms can be understood in a more general sense beyond time constants from the classical experimental paradigms, as Webster and MacLeod discussed in their seminal review paper^33^. A convincing example is the other race effect^37^, which provides clear evidence for adaptation effects with time constants in the range of long-term memory. The reduction of this other race effect in people who spend some time (months or years) in other-race countries shows adaptation effects with intermediate time constants^34^.

The current two experimental conditions differ in the range of stimulus variants and thus in the “average happiness” across stimuli within conditions. The average happiness-value from one condition may have adapted the participants perceptual system, resulting in the shift of the psychometric function found in our data. However, the order of conditions was randomized across subjects, i.e. half of the subjects started with the Full-Range Condition and the other half with the Half-Range Condition. We should thus observe opposite adaptation effects for the two half groups but we did not.

### About the potential role of serial dependency effects

Serial dependence is a mechanism, which assures that our perception of the physical environment from one moment to another can be regarded as continuous. In fact, positive serial dependence would assimilate the information of the previous and present stimulus to build up a perception^38^,^39^. It has been described for facial identity^40^ and attractiveness^41^. Very recently, a negative serial dependency effect has been described for the emotional face perception^42^. In this case the perception of the previous emotional face expression wouldn’t be integrated in the current percept, but rather has contrastive effects on the current percept, probably to maximize the detection of naturally quick changes of expressions^41^. In the Half-Range Condition of the present experiment the net number of face stimuli with downwards pointing endpoints of the mouth curvature is larger than in the Full-Range Condition. This may have increased the happy-percept-probability of some intermediate stimuli, compared to the Full-Range Condition. Negative serial dependency may thus explain the sigmoid shift between conditions.

### About the potential role of anchoring

Another possible explanation for the observed shift of psychometric functions may be the following: Each stimulus sequence was presented 30 times and participants may have learned about the range of stimulus variants and calibrated their internal happy-sad scale to this exogenously presented range. We tested this hypothesis by taking the participants’ mean across the first 5 stimulus sequences and comparing them with the participants’ mean across the last 5 sequences. No significant difference between the sigmoidal functions of the first and the last 5 sequences within conditions was found, although there is some tendency visible for a difference in the slope variable. Most importantly, the shift of the sigmoidal functions between the two conditions is already visible in the average of the first five sequences (Fig. 6) and statistically indicated. The proposed calibration of the endogenous happy-sad scale must thus have taken place surprisingly rapid, possibly during the first sequence of stimulus presentation. Such a quick recalibration is reminiscent of anchoring effects in the range of rating scales^43^,^44^, where one stimulus or a given stimulus range can serve as an anchor in the sense of a standard reference for “stimuli under consideration”^43^. Anchoring effects have been shown at lower and higher complexity levels of sensory input^44^,^45^. In particular it has been shown that one or few initial stimulus examples are enough to induce an anchoring effect^45^.

Whether the sigmoid shift can be explained by adaptation or serial dependency on the sensory level, or anchoring on the decision level has to be shown in further experiments.

### About Ambiguity

Ambiguity of a piece of sensory information means that two or more interpretations are possible. The ambiguity of the classical visually ambiguous figures, like the famous Necker cube^2^ or Rubin’s famous Vase/Face figure^3^, is mainly binary in nature. Typically the perceptual system oscillates between two most probable interpretations (two different 3D configurations of the Necker cube; or either a vase of a face in Rubin’s figure), although other – less probable – interpretations may also exist. Things are more complicated in the case of higher-level ambiguity, e.g. the emotional expression of face stimuli. As in the current experiments, often a number of equidistant stimulus variants along one certain feature dimension are created, resulting in unambiguous perceptual interpretations at the extreme points (like happy and sad face expressions in the current study). The presentation of the stimulus sample is typically combined with a binary forced choice task (e.g. happy vs. sad percept). The stimulus at the mid point along the feature axis may then be labelled as ambiguous, given that the probabilities of the two response options are both at about 50%. However, the binary task hides the possibility of other perceptual interpretations, like that of a neutral face. In terms of perceptual probabilities it may thus be necessary to differ between binary (e.g. Necker cube) and non-binary situations (e.g. face morphing along the happy-sad or gender axes), when using the term “ambiguous”.

Da Vinci’s Mona Lisa is special in this context, because there seems to be a general agreement about the painting’s ambiguity. Assuming this, it is unlikely that any of our variants has been perceived as neutral, although we did not ask explicitly about that.

## Conclusions

Given the present ranges of Mona Lisa variants, Leonardo da Vinci’s original was always perceived as happy. We were able to identify Mona Lisa variants in our selection of stimuli, with roughly equal numbers of happy and sad face identifications and labelled them as ‘ambiguous’. Whether perception of them is really ambiguous, in the sense of classical binary ambiguous figures, has to be shown in future experiments. The identity of the most ‘ambiguous’ stimulus variant in our study depended on and changed with the underlying range of happy and sad stimulus variants in the two experimental conditions. Our data demonstrate that visual perception is highly adaptive and a recalibration of a complex, cognitive feature, like the emotional face expression, seems to take place rapidly within the first few exposures to the whole stimuli range.

The present data suggest that “ambiguity” along the happy-sad axis of emotional face expressions is not the central feature making da Vinci’s painting as famous as it is, because perception of da Vinci’s original stayed happy across the two experimental conditions. However, perception of and reaction to emotional face content is relative and strongly depends on the stimulus context.

An interesting next step would be, to quantify observers’ perception of da Vinci’s Mona Lisa presented in isolation, without any adapting influence nor any reference system of happier and sadder stimulus variants in the immediate spatio-temporal vicinity. In this case the number of observers need to be increased and each observer should be asked only once, simply because the perception process obviously changes the perceptual system – another interesting analogy of cognitive functions to a core feature in quantum physics^46^. Further, other spatial and temporal contexts of the individual observer need to be controlled as well.

## Additional Information

**How to cite this article**: Liaci, E. *et al*. Mona Lisa is always happy – and only sometimes sad. *Sci. Rep.* **7**, 43511; doi: 10.1038/srep43511 (2017).

**Publisher's note:** Springer Nature remains neutral with regard to jurisdictional claims in published maps and institutional affiliations.

## Figures and Tables

**Figure 1:**
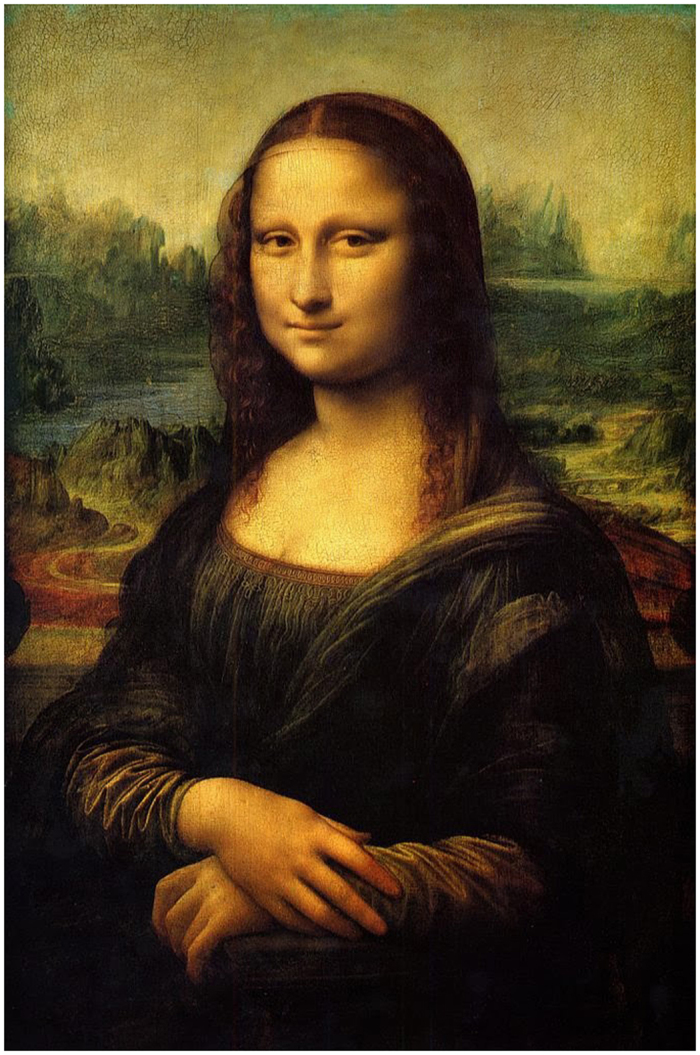
“Mona Lisa” by Leonardo da Vinci (around 1503–1516)^25^.
